# Bacterial Communities of Diatoms Display Strong Conservation Across Strains and Time

**DOI:** 10.3389/fmicb.2018.00659

**Published:** 2018-04-06

**Authors:** Gregory Behringer, Michael A. Ochsenkühn, Cong Fei, Jhamal Fanning, Julie A. Koester, Shady A. Amin

**Affiliations:** ^1^Marine Microbial Ecology Lab, Biology Program, New York University Abu Dhabi, Abu Dhabi, United Arab Emirates; ^2^College of Resources and Environmental Science, Nanjing Agriculture University, Nanjing, China; ^3^Department of Biology and Marine Biology, University of North Carolina at Wilmington, Wilmington, NC, United States

**Keywords:** phytoplankton–bacteria interactions, diatoms, microbial interactions, phytoplankton microbiome, marine microbial ecology, microalgae

## Abstract

Interactions between phytoplankton and bacteria play important roles in shaping the microenvironment surrounding these organisms and in turn influence global biogeochemical cycles. This microenvironment, known as the phycosphere, is presumed to shape the bacterial diversity around phytoplankton and thus stimulate a diverse array of interactions between both groups. Although many studies have attempted to characterize bacterial communities that associate and interact with phytoplankton, bias in bacterial cultivation and consistency and persistence of bacterial communities across phytoplankton isolates likely impede the understanding of these microbial associations. Here, we isolate four strains of the diatom *Asterionellopsis glacialis* and three strains of the diatom *Nitzschia longissima* and show through metabarcoding of the bacterial 16S rDNA gene that though each species possesses a unique bacterial community, the bacterial composition across strains from the same species are highly conserved at the genus level. Cultivation of all seven strains in the laboratory for longer than 1 year resulted in only small changes to the bacterial composition, suggesting that despite strong pressures from laboratory culturing conditions associations between these diatoms and their bacterial communities are robust. Specific operational taxonomic units (OTUs) belonging to the Roseobacter-clade appear to be conserved across all strains and time, suggesting their importance to diatoms. In addition, we isolate a range of cultivable bacteria from one of these cultures, *A. glacialis* strain A3, including several strains of *Shimia marina* and *Nautella* sp. that appear closely related to OTUs conserved across all strains and times. Coculturing of A3 with some of its cultivable bacteria as well as other diatom-associated bacteria shows a wide range of responses that include enhancing diatom growth. Cumulatively, these findings suggest that phytoplankton possess unique microbiomes that are consistent across strains and temporal scales.

## Introduction

Interactions between phytoplankton and bacteria are some of the most important relationships in aquatic environments (Cole, 1982; Azam and Malfatti, 2007; Seymour et al., 2017). Bacteria are inherently dependent on the photosynthetic phytoplankton to acquire organic carbon needed to sustain their growth (Field et al., 1998; Falkowski et al., 2008); in turn phytoplankton rely on bacteria to remineralize organic matter back to inorganic substituents that ultimately support algal growth (Cho and Azam, 1988; Worden et al., 2015). More recent research has shown that interactions between both groups are complex, involving the exchange of cofactors, micronutrients, macronutrients, proteins, and signaling molecules. These exchanges result in mutualistic, commensal, competitive, and antagonistic interactions that can lead to the demise or success of interacting species (Amin et al., 2012; Kazamia et al., 2016; Seymour et al., 2017). For example, more than 170 microalgal species out of 326 tested species cannot synthesize vitamins B_1_, B_7_, and B_12_, yet require it to grow (Croft et al., 2005; Tang et al., 2010). Many bacteria fulfill this requirement by synthesizing vitamins in exchange for algal photosynthates. The ubiquitous marine bacterium *Ruegeria pomeroyi* alleviates vitamin B_12_ limitation of the diatom *Thalassiosira pseudonana* in exchange for organic carbon and sulfur metabolites (Durham et al., 2015, 2017). Some marine bacteria increase the bioavailability of iron, a major limiting element in the open ocean (Martin and Fitzwater, 1988), to their algal partners by producing photolabile iron-binding chelates (Amin et al., 2009). Phytoplankton–bacteria interactions can also involve cell cycle manipulations. For example, bacteria belonging to the *Roseobacter* clade convert tryptophan secreted by phytoplankton to the hormone indole-3-acetic acid (IAA). In turn, IAA enhances the cell division of algal cells, its photosynthetic machinery, and potentially its carbon output to the bacteria (Amin et al., 2015; Segev et al., 2016). Other bacteria manipulate algal growth by producing proteins that lyse algal cells or unknown factors that arrest algal cell division (Paul and Pohnert, 2011, 2013; Seyedsayamdost et al., 2011; van Tol et al., 2017).

Algal–bacterial interactions are hypothesized to occur in the microenvironment surrounding algal cells known as the phycosphere (Bell and Mitchell, 1972; Amin et al., 2012; Seymour et al., 2017), the aquatic analog of the rhizosphere in plants (Mendes et al., 2011). The phycosphere is a region characterized by relatively high concentrations of organic molecules released by algal cells relative to bulk seawater (Biddanda and Benner, 1997). Bacteria colonize the phycosphere either using chemotaxis, random encounter with algal cells or via vertical transmission (Seymour et al., 2017). It is becoming apparent that though these interactions occur at the scale of individual cells, they have major implications at the ecosystem level. Understanding the mechanisms of these interactions and the major players (bacteria and phytoplankton) that mediate these relationships is essential to modeling and predicting changes to the marine environment.

In order to understand interactions in the phycosphere, we must first determine the types of bacteria that associate with different species of phytoplankton and their abundance. Most studies examining bacterial association with phytoplankton rely mostly on cultivation of bacteria from algal cultures or algal blooms and on metagenomic sequencing of algal blooms. For example, marine bacteria that have been consistently isolated from diatom cultures and diatom-dominated blooms belong to a small number of genera relative to total bacterial genera found in seawater (Sapp et al., 2007; Amin et al., 2012; Baker and Kemp, 2014). Similar examples have been shown in other phytoplankton lineages (Biegala et al., 2002; Green et al., 2004; Hasegawa et al., 2007; Eigemann et al., 2013). For example, two coccolithophore species have also been shown to harbor specific types of bacteria across several cultures (Green et al., 2015) while specific bacteria have been shown to associate with certain dinoflagellate species (Green et al., 2004; Bolch et al., 2011). Blooms of the colonial cyanobacterium *Trichodesmium* appear to also harbor a core microbiome (Frischkorn et al., 2017). However, some microalgal species do not appear to harbor a core set of bacteria. For example, 13 different cultures of the green alga *Ostreococcus tauri* contain varying bacteria with no apparent microbiome at the genus level (Abby et al., 2014). Cumulatively, these studies demonstrate that although there is a general tendency to find specific species of bacteria that associate with phytoplankton, more work is needed to truly define phytoplankton microbiomes.

Unlike plants or mammals, defining the microbiome of phytoplankton is difficult given that they are unicellular organisms that grow in aquatic, dilute environments. Cultivation studies are inherently biased since most marine bacteria cannot be isolated and maintained through existing culturing techniques (Rappé and Giovannoni, 2003). Isolation and culturing of microalgae and their associated bacteria in the laboratory for prolonged periods before identification of bacteria is also problematic as it is hypothesized that under nutrient-rich laboratory conditions, the microbiome may undergo major changes in composition. Metagenomic sequencing of algal blooms also poses a problem since other bacteria in seawater could be misidentified as algal-associated.

Although previous studies have highlighted important taxa that interact with many phytoplankton species, our knowledge of the abundance and persistence of these bacterial taxa with their algal host either in the environment or in laboratory cultures is lacking. In addition, the occurrence of specific species of bacteria across strains of the same phytoplankton species, which would suggest ‘intimate’ associations between an alga and its ‘microbiome,’ has been largely unexamined. To address some of these limitations, we isolated multiple strains of the same species of phytoplankton and cultured them in the laboratory in order to compare the microbial communities across strains and time. We isolated single cells of the diatoms *Asterionellopsis glacialis* and *Nitzschia longissima* from different locations in the Arabian Gulf around the coastline of Abu Dhabi. Shortly after isolation, we characterized the microbiomes of all diatom isolates and continued to monitor changes to these microbiomes over the course of one year. Using *A. glacialis* as a model for studying algal–bacterial interactions, we cultivated bacteria from this diatom and cocultured them to find if these bacteria play any role in the physiology or life cycle of the diatom.

## Materials and Methods

### Diatom Isolation, Maintenance, and Identification

Single diatom cells (or chains) were isolated in October 2015 and July 2016 from different locations in the Arabian Gulf near the Abu Dhabi Coast, United Arab Emirates by dilution of a small volume (<0.2–0.5 μL) in sterile f/2+Si medium (Ryther and Guillard, 1962). Volumes isolated from seawater were observed using light microscopy prior to dilution with sterile media to ensure a single diatom cell/chain was present in the sample. Isolates were identified using light microscopy (Leica DM IL LED, Wetzlar, Germany) and scanning electron microscopy (SEM) using a 5-kV beam and the secondary electron detector for platinum-palladium coated (12 nm) samples (Phillips XL 30S FEG SEM, FEI Inc., Hillsboro, OR, United States). Putative taxonomic identities were assigned based on morphological characteristics as well as partial sequencing of the 18S rDNA and the partial sequencing of both internal transcribed spacer (ITS) regions and 5.8S rDNA.

Batch cultures were maintained in sterile borosilicate culture tubes (Fisher, Hampton, NH, United States) containing 25-mL of sterile f/2+Si media. All diatom cultures were grown in semi-continuous batch cultures (Brand et al., 1981) in algal growth chambers (Percival AL-30L2 and AL-36L4) at 22°C, 125 μE m^-2^ s^-1^, and 12:12 light/dark cycle. All cultures were transferred approximately every 7 days by inoculating 25 mL fresh media with 50 μL of culture. Although the growth rate of each culture varied over the course of the experiment, on average cultures were transferred 30 times between each time point for bacterial profiling. Light flux was measured using a QSL-2100 PAR Sensor (Biospherical Instruments Inc., San Diego, CA, United States). Diatom growth was monitored initially by direct cell counts using a Sedgwick rafter (Pyser-SGI Ltd., Kent, United Kingdom) and once a linear relationship between cell number and *in vivo* fluorescence was established, by measuring relative fluorescence units (RFUs) using a 10-AU fluorimeter (Turner Designs, San Jose, CA, United States). Specific growth rates (μ) were calculated from the linear regression of the natural log of *in vivo* fluorescence versus time during the exponential growth phase of cultures. Standard deviation of μ was calculated using μ values from biological replicates (*n* = 6 unless otherwise indicated) over the exponential growth period.

### Diatom DNA Isolation and Phylogeny

For DNA isolation, log-phase diatom cultures were vacuum filtered onto a 47 mm 3-μm cyclopore membrane filters (Whatman) and flash frozen in liquid nitrogen. Subsequently, DNA was extracted either with the PureLink Plant Total DNA Purification kit (Invitrogen, Life Technologies) directly or cells were lysed by repeated freeze/thaw cycles in liquid nitrogen, incubation at ∼90°C in a water bath followed by pulsed bead beating (BioSpec, Bartlesville, OK, United States) with zirconium ceramic oxide bulk beads (Thermo Fisher Scientific) in “Nuclei Lysis Buffer” (Promega, Madison, WI, United States). The resulting lysate was processed via the Wizard genomic DNA Purification Kit using the plant tissue protocol (Promega). Targeted DNA loci were then amplified using Q5 High-Fidelity 2X Master Mix (New England Biolabs, Ipswich, MA, United States) and the primer pair ITS1-FWD TCCGTAGGTGAACCTGCGG, and ITS4-REV TCCTCCGCTTATTGATATGC (White et al., 1990) and 18S primers A: AACCTGGTTGATCCTGCCAGT and B: TGATCCTTCTGCAGGTTCACCTAC (Medlin et al., 1988). PCR cycle conditions for ITS were as follows: an initial 94°C for 1 min followed by 94°C for 1 min, 51°C for 1 min, 72°C for 1 min for 35 cycles, and a final incubation at 72°C for 8 min. PCR cycle conditions for 18S rDNA were as follows: an initial 94°C for 2 min followed by 95°C for 1 min, 58°C for 45 s, 72°C for 1 min for 35 cycles, and a final incubation at 72°C for 5 min. ITS products were size-selected using gel electrophoresis on a 1% agarose gel, and excised bands were purified with the Wizard SV Gel and PCR Clean-Up kit (Promega). DNA was stored at -20°C until Sanger sequenced (GENEWIZ, South Plainfield, NJ, United States). For 18S sequencing, PCR reactions were cleaned with ExoSAP (Applied Biosystems) and Sanger sequenced (NC State University, Genome Sciences Laboratory, Raleigh, NC, United States). The NCBI BLASTn suite^1^ (Altschul et al., 1990) was used to assign taxonomy to all consensus contigs.

Consensus sequences for ITS were aligned in MAFFT version 7 (Katoh and Standley, 2013). Alignments were then exported to BEAUti (Bayesian Evolutionary Analysis Utility Version v1.8.4) (Drummond et al., 2012). The Markov chain Monte Carlo (MCMC) was set in BEAUti with 100,000,000 generations. Resulting XML files were analyzed by BEAST (Bayesian Evolutionary Analysis Sampling Trees Version v1.8.4) (Drummond et al., 2012) and post chain termination Markov Chain convergence was assessed using Tracer (MCMC Trace Analysis Tool Version v1.6.0). Consensus trees were analyzed using FigTree v1.4.3. A supplemental maximum-likelihood (ML) inference was also run for diatom loci using RAxML 7.3.0 (Stamatakis, 2006) to complement the findings of the above Bayes analysis, as the two can sometimes conflict (Beerli, 2006). A partitioned dataset was run under the model parameter “GTRGAMMA,” and rapid bootstrap analysis (Stamatakis et al., 2008) was performed with 1,000 bootstraps.

### Bacterial DNA Isolation

Twenty days after isolation, 10 mL of each culture with RFU ∼12–16 (mid-to-late exponential phase) were gently vacuum filtered onto a 25 mm 0.2-μm polycarbonate membrane filters (Whatman, NJ, United States). All filters were flash frozen and stored at -80°C for later processing. This process was repeated approximately every 6 months (200 days and 400 days) with cultures at the same cell densities as the original filtration mainly to avoid variations of the bacterial community as a function of culture density. For bacterial DNA isolation, 25% of each filter was excised using sterile scissors and DNA was isolated using E.Z.N.A. bacterial DNA kit (OMEGA Bio-tek, Norcross, GA, United States) according to the manufacturer’s protocol. DNA was stored at -80°C until further processing.

### 16S rDNA Profiling of Bacteria

All samples were amplified and sequenced by the University of Illinois’s Roy J. Carver High-Throughput Sequencing and Genotyping Unit (Urbana, IL, United States). In brief, DNA concentrations were measured via a Qubit instrument (Thermo Fisher) and diluted to a final concentration of 2 ng/μl. Reaction mixes contained the Fast Start High Fidelity PCR System (Roche, Basel, Switzerland) and 20x Access Array loading reagents (Fluidigm, San Francisco, CA, United States) per the manufacturer’s protocol. For 16S rDNA amplification, the following bacterial primers were used: 515FB-FWD: GTGYCAGCMGCCGCGGTAA and 806RB-REV: GGACTACNVGGGTWTCTAAT. PCR was performed using standard conditions (Parada et al., 2016). PCR amplicons were size selected on a 2% agarose E-gel (Thermo Fisher) and purified via a gel extraction kit (Qiagen, Hilden, Germany). Amplicon sizes were then verified post purification using a Bioanalyzer (Agilent, Santa Clara, CA, United States). Illumina reads of the bacterial communities from all samples were deposited in the short read archive (Accession No. SRP132349).

Recovery of Archaeal amplicons from filtrate samples was also attempted using the same method as for bacteria except for the Fluidigm protocol, the following primer pairs were used: Arch349F (5′-GYGCASCAGKCGMGAAW-3′) and Arch806R (5′-GGACTACVSGGGTATCTAAT-3′) (Takai and Horikoshi, 2000). No archaeal amplicons were recovered using this protocol.

### Phylogenetic Analysis of Bacteria

For Illumina sequencing, samples were denatured and spiked with a 20% non-indexed PhiX control library (Illumina) and loaded onto a MiSeq V2 flowcell at a final concentration of 8 pM. Libraries were then paired-end sequenced (2x250 bp). Generated ^∗^.bcl files were converted into demultiplexed compressed FASTQ files. The “FastX-Toolkit” (Hannon Lab^2^) was employed for quality checks and other metrics. Sequences were analyzed using the Mothur platform (Schloss et al., 2009). After contig alignment and trimming, identical sequences were merged using the ‘unique.seqs’ command to save computation time, and the command ‘count.seqs’ was used to keep a count of the number of sequences over samples represented by the remaining representative sequence. Rare sequence reads were removed (*n* < 10) and the remaining sequences were aligned against the SILVA database (release 128) (Quast et al., 2012). Chimeric sequences were removed using UCHIME as implemented in MOTHUR (Schloss et al., 2009). Chloroplast, mitochondrial, eukaryotic, and unknown reads were also removed. Sequences were classified against Greengenes using bootstrapping of 60, and the sample compositions were compared on the family level (Caporaso et al., 2010). For further analyses, a 97% similarity cut-off level was chosen to obtain operational taxonomic units (OTUs) after trimming, singleton and chimera removal and chloroplast filtering.

Both Bayesian Markov chain Monte Carlo (MCMC) and ML analysis methods were used to analyze phylogeny of bacterial isolates. MRBAYES 3.1.2 (Ronquist and Huelsenbeck, 2003) was used for Bayesian MCMC analysis of 16S rDNA with the GTR model (Lanave et al., 1984). The ML analysis was performed using PhyML 3.0 (Guindon et al., 2010) and an automatic model was selected by SMS (Lefort et al., 2017).

### Isolation of Cultivable Bacteria From *A. glacialis*

To isolate individual bacterial colonies, several non-axenic A3 cultures in the exponential growth phase were serially diluted, and plated (75–100 μL aliquots) onto four different seawater agar plates (15.0 g/L agar in 0.2-μm filtered seawater) that contained either 2.0g/L casamino acids (Thermo Fisher), 2.0g/L dibasic sodium succinate (Sigma-Aldrich), 3.0 g/L D-(+)-glucose (Sigma-Aldrich), or plain seawater. In addition, Marine Broth 2216 (HIMEDIA, Mumbai, India) dissolved in MQ-H_2_O was also used as an additional culturing plate. Plates were incubated at 25°C and single colonies were picked with sterile toothpicks and cultured in the respective liquid media types. Liquid cultures were grown overnight in a Stuart Orbital SI 600 shaker at 25°C and a glycerol stock was preserved for later experiments and bacterial identification. For bacterial identification, 16S rDNA gene was amplified using universal primers (27F, 1492R) as previously described (Amin et al., 2015). Amplicons were Sanger sequenced (GENEWIZ, South Plainfield, NJ, United States). All Sanger sequences of bacterial 16S rDNA were deposited in GenBank (Accession Nos. MG488233–MG488271).

### Axenic *A. glacialis* Culture Generation

Axenic cultures were generated as described previously (Shishlyannikov et al., 2011) with some modifications. In brief, approximately 25 ml of a late-exponential phase growing diatom culture was gravity filtered onto 0.65 μm pore-size polycarbonate membrane filter (Millipore). Cells were quickly rinsed with sterile f/2+Si media. Using sterile tweezers, the filter was carefully removed from the filtration unit and washed for ∼1 min in sterile media containing 20 mg/ml Triton X-100 detergent to remove surface-attached bacteria. The filter was discarded after re-suspension of cells by gentle shaking in sterile detergent-free media. Cells were again gravity filtered onto a fresh 0.65-μm pore-size polycarbonate membrane filter and rinsed with sterile media. Subsequently, cells were washed off the filter by gentle shaking into sterile media containing a suite of antibiotics (per milliliter: 50 μg streptomycin, 66.6 μg gentamycin, 20 μg ciprofloxacin, 2.2 μg chloramphenicol, and 100 μg ampicillin). Cells were incubated in antibiotic-containing media for 48 h under regular growth conditions. Finally, 0.5–1.0 mL of antibiotics-treated cells were transferred to antibiotic-free media. Cultures were regularly monitored for bacterial contamination by checking for bacterial growth in Zobell marine broth (ZoBell, 1941) in addition to filtering 2–3 mL of exponential-phase growing culture and using Sybr Green I (Invitrogen) staining and epifluorescence microscopy (Nikon Eclipse 80i) as described previously (Lunau et al., 2005).

### Coculture Experiments

Bacterial isolates were plated before each experiment on marine agar and were grown from single colonies in marine broth overnight (30°C, 150 rpm). Bacteria were centrifuged (3,500 ×*g* for 5 min), washed twice with sterile f/2+Si, and inoculated into sterile, fresh 25-mL tubes containing f/2+Si media at a final cell density of ∼1 × 10^4^ cells/mL. Axenic diatom cultures were acclimated for at least three transfers using semi-continuous batch cultures (Brand et al., 1981). Cultures were considered acclimated if the growth rate of three consecutive transfers of triplicate cultures did not vary by more than 15%. Subsequently, cultures were inoculated into the same tubes at a final concentration of ∼5,000 cells/mL. Cocultures were incubated as described above for diatoms. Statistical analysis of coculture growth rates were performed using omnibus ANOVA testing and *post hoc*, pairwise comparisons were generated from Tukey Honest Significance Difference tests.

## Results

### Diatom Isolation

**FIGURE 1:**
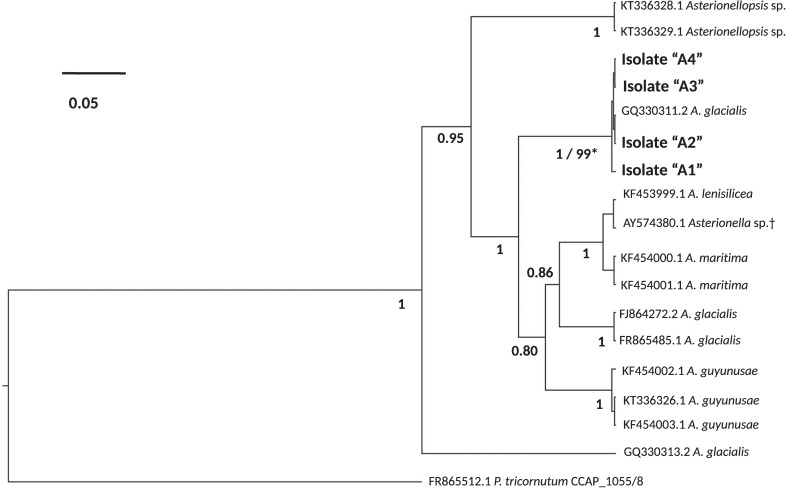
Phylogeny of the diatoms *Asterionellopsis glacialis. A. glacialis* Bayesian phylogeny was constructed using internal transcribed spacer 1 (ITS1), 5.8S rRNA gene and internal transcribed spacer 2 (ITS2) of four isolates (A1–A4). Main nodes are labeled with posterior probability values (0.0–1.0) and clade for our isolates is also supported by a maximum-likelihood (ML) bootstrap value (0–100). Scale bar represents nucleotide substitutions per site. ^†^Current NCBI nomenclature. However, recent literature suggests erroneous naming in NCBI and prescribes assignment to the *Asterionellopsis* genus (Moniz and Kaczmarska, 2011).

To examine the bacterial composition of diatoms, the persistence of these microbiomes over culturing time in the laboratory and their conservation across different strains of the same species, we isolated four single-cell isolates of a putative *Asterionellopsis* sp. (A1, A2, A3, and A4) and three isolates of a putative *Nitzschia* sp. (N1, N2, and N3). To confirm the identities of these diatoms, the 18S rDNA gene was Sanger sequenced. 18S rDNA sequences from *Asterionellopsis* spp. showed the strongest similarity to the *A. glacialis* CCAP 1009/3 (FR865485) 18S rDNA gene in NCBI. *A. glacialis* sensu lato has been shown to possess a wide cryptic diversity that encompasses at least five separate species, *A. glacialis*, *A. tropicalis*, *A. guyunusae, A. maritima*, *A. lenisilicea*, and *A. thurstonii*, using ITS1, 5.8S rRNA, and ITS2 sequences (Franco et al., 2016). To confirm that our isolates were indeed *A. glacialis* we further sequenced the ITS1, 5.8S rRNA, and ITS2 regions of all four *Asterionellopsis* isolates. Phylogeny based on these sequences showed that indeed all four isolates belonged to the *A. glacialis* clade (**Figure 1**).

**FIGURE 2:**
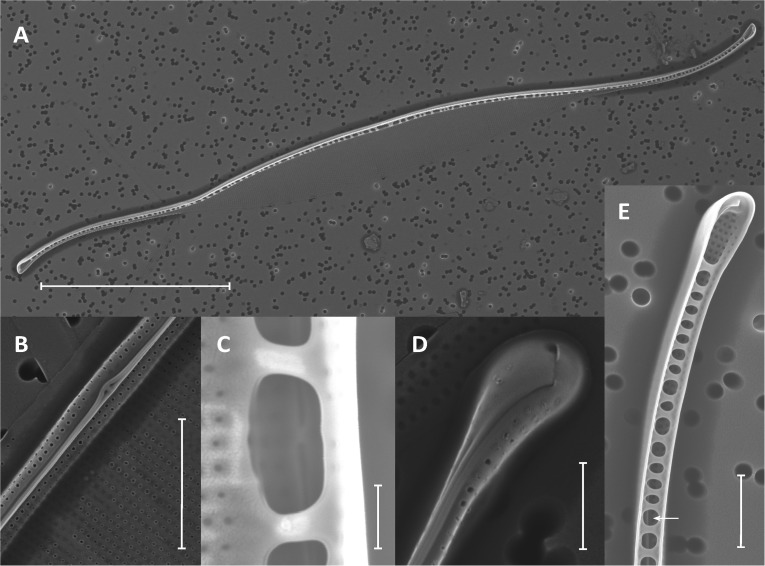
Morphological identification of *Nitzschia longissima* using SEM. **(A)** Interior of a single valve with eccentrically placed, rib-like fibulae and lanceolate ends; scale bar = 20 μm. **(B)** Exterior of the elevated and eccentrically placed raphe and raphe ends at the midpoint of the valve (main body of valve is collapsed); scale bar = 2 μm. **(C)** Interior raphe canal and ends between and behind two fibulae; scale bar = 500 nm. **(D)** Exterior of the apical end of the valve and terminal raphe fissure; scale bar = 1 μm. **(E)** Interior view of raphe canal (arrow) and helictoglossa (terminal end of valve canal) with fused fibulae; scale bar = 2 μm.

Like *Asterionellopsis*, the taxonomic status of *N. longissima* remains unclear due to variation in species descriptions (Kaczmarska et al., 2007). The 18S rDNA sequences from our *Nitzschia* spp. showed the strongest similarity to *N. longissima* (AY881968). Analysis of the ITS regions and 5.8S rRNA sequence of N1, N2, and N3 failed to unequivocally show that these isolates belong to *N. longissima* since NCBI contains no ITS sequences from this species. Therefore, we relied on morphological evidence using SEM to provisionally assign our *Nitzschia* sp. isolates to *N. longissima* (**Figure 2**). Cells were lanceolate in shape with pronounced tapering at the ends (**Figure 2A**) and each contained two elongated chloroplasts. The morphological characteristics determined from SEM include an average of 15 ± 0.63 (SD) fibulae and 51.75 ± 1.28 striae per 10 μm (*N* = 6 and 8 valves, respectively) (**Figures 2B–E**). These characteristics corroborate that N1–N3 likely belong to *N. longissima* (Kaczmarska et al., 2007).

To characterize the bacterial communities of these isolates and assess their stability over laboratory culturing time, non-axenic diatoms were filtered at different time points, DNA was extracted and the bacterial 16S rDNA was amplified and partially sequenced using the Illumina MiSeq platform as described in the Materials and Methods. For *A. glacialis*, cultures A2 and A3 were sampled at 20, 200, and 400 days after isolation while cultures A1 and A4 were sampled only at 20 and 200 days after isolation. All *N. longissima* cultures (N1–N3) were sampled at 20, 200, and 400 days after isolation (**Table 1**).

**Table 1:** Overview of Illumina MiSeq reads and OTUs recovered from each diatom culture.

Species	Sample name	Site of isolation	Isolation date	Time in culture (days)	Total reads	Number unique OTUs
*A. glacialis*	A1	Lat: 24.5177	10/2015	20	158,213	9
		Long: 54.3383		200	192,670	8
	A2	Lat: 24.5941	10/2015	20	167,785	8
		Long: 54.4501		200	306,132	11
				400	133,910	8
	A3	Lat: 24.5941	10/2015	20	158,512	9
		Long: 54.4501		200	157,125	7
				400	171,932	9
	A4	Lat: 24.5970	6/2016	20	97,633	9
		Long: 54.4940		200	114,888	8
						
*N. longissima*	N1	Lat: 24.5941	10/2015	20	114,142	10
		Long: 54.4501		200	108,087	8
				400	148,584	7
	N2	Lat: 24.5177	10/2015	20	167,651	8
		Long: 54.3383		200	223,772	12
				400	300,069	8
	N3	Lat: 24.5177	10/2015	20	126,532	8
		Long: 54.3383		200	216,973	10
				400	139,197	6

### *A. glacialis* Bacterial Community

**FIGURE 3:**
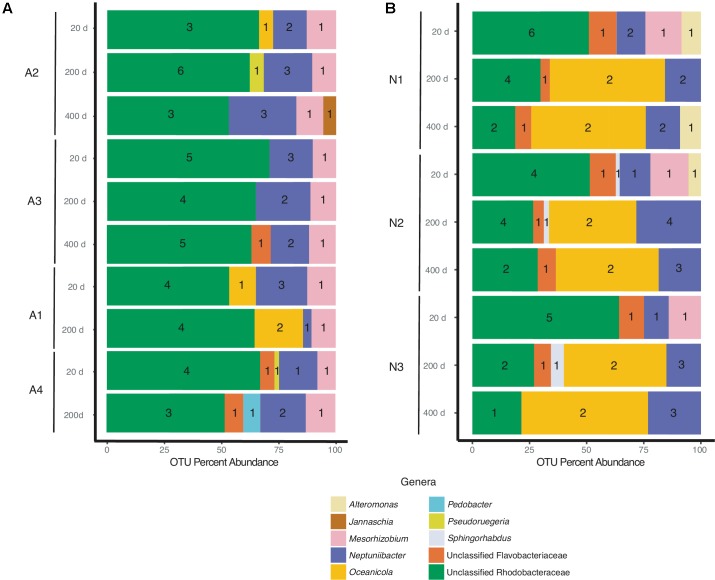
Bacterial composition of **(A)** *A. glacialis* (A1, A2, A3, and A4) and **(B)** *N. longissima* (N1, N2, and N3) across culturing time in the laboratory at the genus level. Numbers inside colored bars represents unique number of OTUs for a specific genus.

Despite differences in the timing of sampling and isolation of the *A. glacialis* strains, all four strains displayed strong conservation of their microbiome across cultures and time with small differences at the genus level. Twenty days after isolation, the microbiome of all four strains were dominated by unclassified Rhodobacteraceae, an important member of diatom and phytoplankton microbiomes (Mayali et al., 2008; Geng and Belas, 2010; Amin et al., 2012; Goecke et al., 2013), comprising on average ∼60% of all reads (**Figure 3A**). The second most abundant group of bacteria belonged to the *Neptuniibacter* genus, comprising on average ∼20% of all reads. Other genera present include *Mesorhizobium* (Phyllobacteriaceae), a group of rhizobacteria, *Jannaschia* (Rhodobacteraceae), *Pedobacter* (Sphingobacteriaceae) and unclassified Flavobacteriaceae. All cultures were characterized by relatively a small number of OTUs (≤11) (**Table 1** and **Figure 3A**).

As the cultures were propagated in the laboratory, minor differences in the composition of the microbiome were observed. There was a modest reduction in the number of Rhodobacteraceae reads over time in A2, A3, and A4 coupled with an increase in *Neptuniibacter* reads (**Figure 3A**). In general, more significant changes were observed after 400 days relative to 200 days. Mainly, as cultures aged in the laboratory new genera were detected at relatively small percentage of reads (<10%). For example, at 400 days the A2 microbiome included <5% Jannaschia while the A3 microbiome included unclassified Flavobacteriaceae (**Figure 3A**). In addition, at 200 days the A4 microbiome included *Pedobacter* and unclassified Flavobacteriaceae. The variability of some genera over time likely stems from their low abundance in the sample.

### *N. longissima* Bacterial Community

N1–N3 microbiomes contained more diversity at the genus level relative to *A. glacialis* with some genera being conserved between both species. Remarkably, the microbiomes of all isolates had strong similarities to each other at 20 days, suggesting that *N. longissima*, like *A. glacialis*, possesses a core microbiome. N1–N3 reads were dominated by unclassified Rhodobacteraceae (∼55% total OTU abundance) followed by *Neptuniibacter* and *Mesorhizobium* (**Figure 3B**). In addition to genera observed in *A. glacialis*, N1 included *Alteromonas* (Alteromonadaceae) and unclassified Flavobacteriaceae, while N2 and N3 included these two and *Sphingorhabdus* (Sphingomonadaceae) (**Figure 3B**). As seen in *A. glacialis* cultures, percent abundance of some genera changed over time albeit with more drastic changes. For example, *Mesorhizobium* disappeared altogether from all three cultures at 200 days. Rhodobacteraceae in all cultures increased in abundance at 200 days but then decreased again at 400 days with an overall increase when compared to the 20-day time point. Similar to *A. glacialis*, all strains throughout time exhibited relatively low diversity (≤12 OTUs per sample) (**Table 1** and **Figure 3B**). In general, the *A. glacialis* community appeared to decrease in diversity over time while the *N. longissima* community appeared to increase in diversity over the same time frame (**Figure 3B**).

**FIGURE 4:**
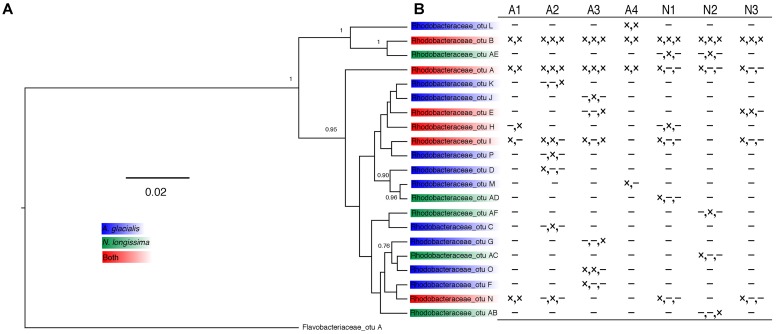
Analysis of Rhodobacteraceae OTUs from *A. glacialis* and *N. longissima*. **(A)** Phylogram showing the relationship between different Rhodobacteraceae OTUs in both diatoms. Posterior probabilities >50 are indicated at the branching points. **(B)** Table showing the distribution of OTUs across samples. Columns represent different strains of each diatom. Rows represent different OTUs with presence (×) or absence (-) indicated for 20 days, 200 days, and 400 days, respectively.

Since Rhodobacteraceae represented the majority of reads from all strains at all time points, further analysis examining the taxonomic relationship between Rhodobacteraceae OTUs and samples were examined. Most OTUs were specific to either *A. glacialis* or *N. longissima* with some variability in their distribution across strains and time (**Figure 4A**). Interestingly, five OTUs were found in both diatoms and two of which (Rhodobacteraceae_otu A and Rhodobacteraceae_otu B) were consistently found across all strains at 20 days and persisted over time for the most part (**Figure 4**). Rhodobacteraceae_otu A is a distant relative of *Pseudoruegeria marinistellae* strain SF-16 and *Shimia biformata* strain CC-AMW-C while Rhodobacteraceae_otu B is a distant relative of *Loktanella koreensis* strain GA2-M3.

### Isolation of Cultivable Bacteria and Coculturing

**FIGURE 5:**
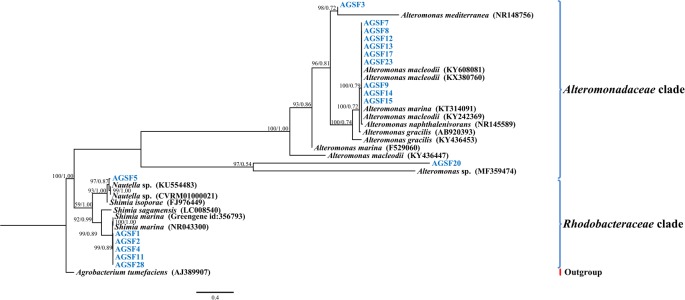
Bayesian phylogenetic tree for the A3 cultivable bacterial 16S rDNA gene. Bacteria isolated from A3 are highlighted in blue. Accession numbers of GenBank sequences are included in parentheses. Family of different clades are indicated on the right. Posterior probabilities and ML bootstrap values are listed next to branches.

In addition to sequencing, we isolated and identified 17 bacteria from the A3 culture at the 20-day time point. In general, cultivable bacterial diversity was low compared to other studies isolating bacteria from diatom cultures (reviewed in Amin et al., 2012). The cultivable bacteria that were recovered belonged to the Alteromonadaceae (*Alteromonas macleodii*, >99% 16S rDNA identity; and *A. marina*, >92% 16S rDNA identity) and Rhodobacteraceae (*Shimia marina*, ≥99% 16S rDNA identity; *Nautella* sp., >97%) representing all isolates (**Figure 5**). Interestingly, the 16S rDNA gene of strains AGSF28, AGSF2, AGSF11, and AGSF4 (**Figure 5**) displayed >95% nucleotide identity to Rhodobacteraceae_otu A (**Figure 4A**), suggesting this OTU may represent a close relative of *Shimia* bacteria in the A3 culture. *Alteromonadaceae* did not represent an important group in the sequencing data highlighting the inherent bias of bacterial cultivation from algal cultures, which has been used in the past to study bacterial association with phytoplankton.

**FIGURE 6:**
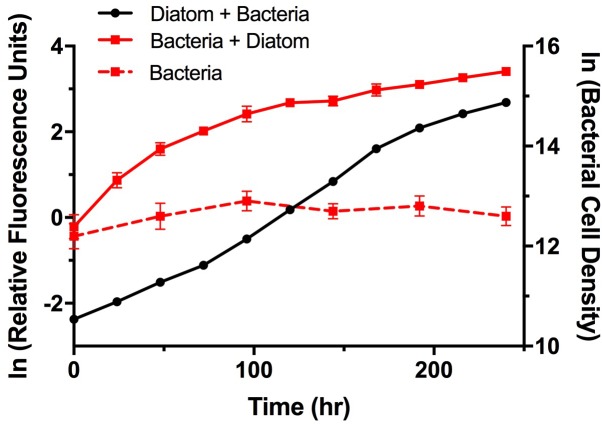
Growth of A3 and its microbiome. Cultures were acclimated as described in the Section “Materials and Methods.” Diatom growth (black circles) with its microbiome was monitored using relative fluorescence and bacterial growth with the diatom (red squares, solid line) or without the diatom (red squares, dashed line) was monitored using microscopy counts of Sybr Green I stained cells. Error bars represent standard deviation (SD) of six cultures.

**FIGURE 7:**
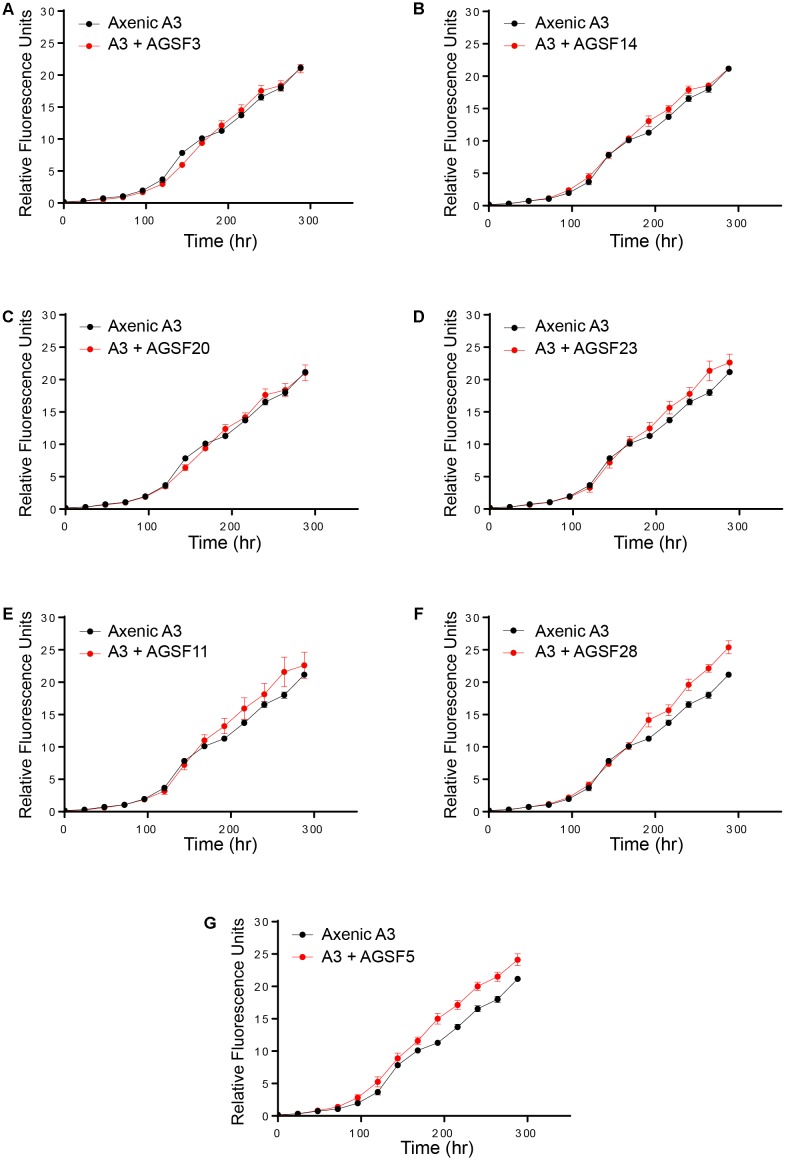
Cocultures of A3 with cultivable bacterial representatives from its microbiome show varying effects of bacterial isolates on the diatom. **(A,C)** Coculture of A3 with two *Alteromonas* sp. isolates. **(B,D)** Coculture of A3 with two *A. macleodii* strains. **(E,F)** Coculture of A3 with two *S. marina* strains. **(G)** Coculture of A3 with a *Nautella* sp. isolate. Error bars represent SD of six cultures.

To examine potential interactions between *A. glacialis* and some of its bacterial consortium members, we first monitored the growth of A3 and its complete microbial consortium. Because the media used for growth, f/2+Si, lack organic carbon besides background organic carbon in seawater (Guillard, 1975), we anticipated that bacterial growth would be dependent on organic exudate excretion from the diatom. Indeed, as A3 grew bacterial cell densities increased from 5.9 × 10^4^ to 3.3 × 10^6^ cells mL^-1^ over the course of 10 days relative to controls without the diatom (**Figure 6**). To examine how different cultivable bacteria influence the growth of *A. glacialis*, A3 was made axenic as described in the Section “Materials and Methods” using a cocktail of antibiotics. Cocultures of A3 and six representative members from **Figure 5** were set up and the growth of the diatom was monitored. When cocultured with *Alteromonas* strains AGSF3, AGSF20 and AGSF23, A3 did not exhibit a significant change in growth rate relative to axenic cultures; however, coculture with *A. macleodii* strain AGSF14 caused a modest increase in growth rate of 5% (**Figure 7** and **Table 2**). Coculture with *S. marina* strains AGSF11 and AGSF28 did not show a significant change in growth rate relative to controls; however, a statistically significant growth enhancement was observed when A3 was cocultured with *Nautella* sp. AGSF5 (**Table 2** and **Figure 7**).

**Table 2:** Change in A3 growth rate (μ) as a function of coculture with different bacteria relative to axenic growth.

Bacterial genus	Name	μ_axenic_ ± SD	μ_co-culture_ ± SD	% Change in μ
*Alteromonas*				
	AGSF14	0.61 ± 0.02	0.65 ± 0.02	5.3**
	AGSF3	0.61 ± 0.02	0.60 ± 0.01	2.8
	AGSF20	0.61 ± 0.02	0.60 ± 0.01	-0.2
	AGSF23	0.61 ± 0.02	0.60 ± 0.01	-1.7
*Nautella*
	AGSF5	0.61 ± 0.02	0.66 ± 0.02	7.4*
*Ruegeria*
	AGSA97^†^	0.72 ± 0.02	0.84 ± 0.03	17^∗^
*Shimia*
	AGSF11	0.61 ± 0.02	0.63 ± 0.02	2.6
	AGSF28	0.61 ± 0.02	0.61 ± 0.01	-0.2
*Sulfitobacter*
	SA11^†^	0.72 ± 0.02	0.88 ± 0.1	22^∗^
*Croceibacter*
	SA60^†^	0.72 ± 0.02	0.57 ± 0.03	-14^∗^

*Standard deviations (SDs) were calculated from six cultures. ^†^Bacteria previously isolated from other diatom cultures. ^∗^p < 0.01 (ANOVA); ^∗∗^p < 0.05 (ANOVA).*

**FIGURE 8:**
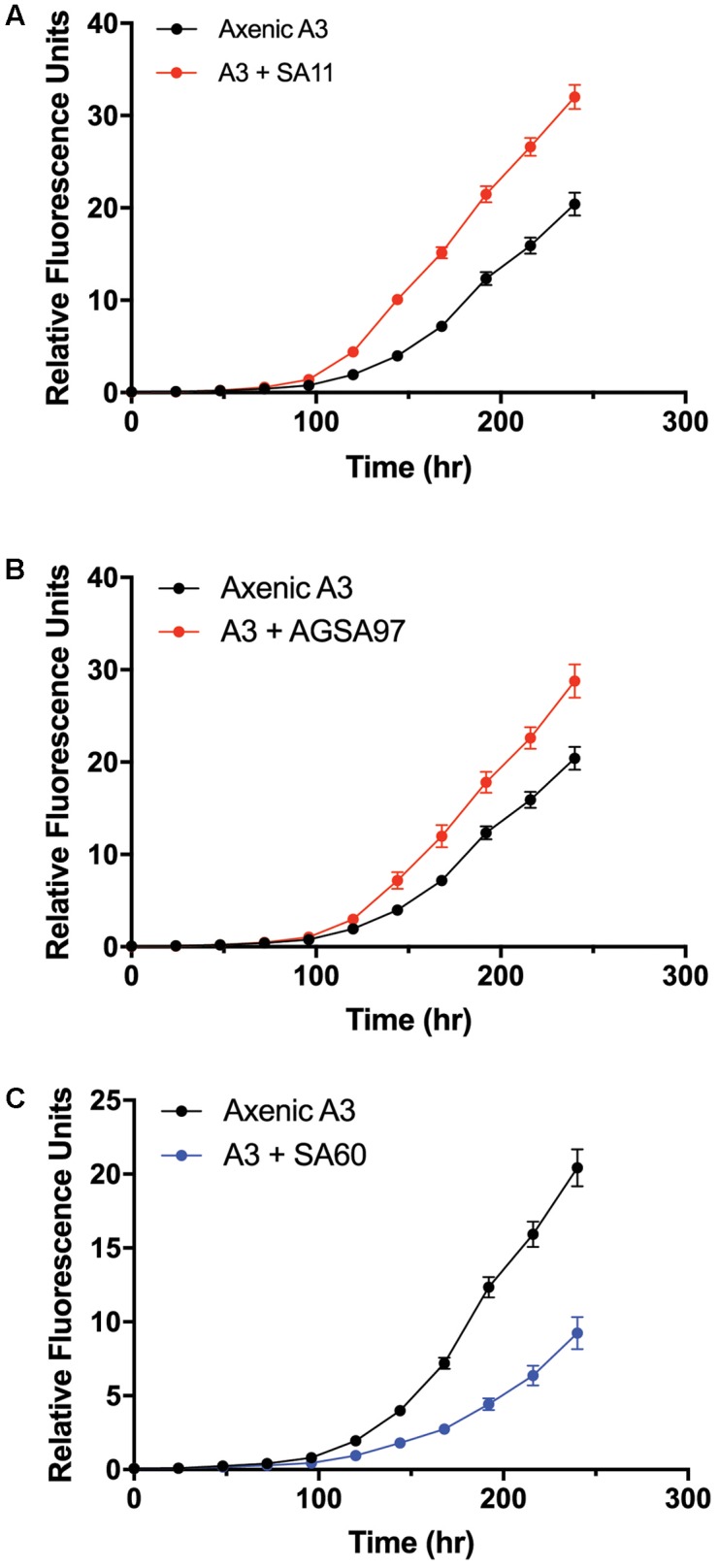
Cocultures of A3 with cultivable bacterial representatives isolated from the diatoms *P. multiseries* and *Asterionella* sp. show either growth enhancement or inhibition on the diatom. **(A)** Coculture of A3 with *Sulfitobacter* sp. SA11 isolated from the pennate diatom *P. multiseries*. **(B)** Coculture of A3 with *Ruegeria* sp. AGSA97 isolated from the pennate diatom *Asterionella* sp. **(C)** Coculture of A3 with *Croceibacter atlanticus* SA60 isolated from *P. multiseries.* Error bars represent SD of six cultures.

To test whether *A. glacialis* responds to other bacteria associated with other diatoms, we cocultured A3 with several strains of bacteria that were previously isolated from other diatoms: *Sulfitobacter* sp. SA11 and *Ruegeria* sp. AGSA97 both belong to Rhodobacteraceae and were previously isolated from the diatoms *Pseudo-nitzschia multiseries* and *Asterionella* sp., respectively. Both bacteria enhanced the growth rate of A3 by 19 and 15%, respectively, relative to axenic A3 (**Figures 8A,B** and **Table 2**). *Croceibacter atlanticus* strain SA60, a bacterium that was isolated from the diatom *P. multiseries* (Amin et al., 2015), was also cocultured with A3 and inhibited its growth relative to axenic controls (**Figure 8C** and **Table 2**).

## Discussion

Research examining interactions between phytoplankton and bacteria typically relies on isolation of bacteria from microalgal cultures that have been maintained in the laboratory for years; however, the relevance of such bacteria to natural bacterial populations encountered by phytoplankton in the environment is not clear. Our study shows that two diatom species possess a microbiome dominated by a relatively small number of unique OTUs (**Table 1**) and that the diatoms maintain high conservation of these microbiomes shortly after isolation from the field and up to more than 1 year in culture under typical laboratory conditions. These results provide preliminary evidence that diatom microbiomes may be similar in composition and do not undergo major changes when cultured in the laboratory. Although analysis of the microbiomes of four *A. glacialis* and three *N. longissima* isolates show small changes particularly after one year of cultivation, overall most of the dominant groups of bacteria persisted beyond 1 year (**Figure 3**). In general, bacterial diversity of the *A. glacialis* strains exhibited a reduction over time while the opposite trend was observed with *N. longissima*, suggesting a fundamental difference between the two diatoms. This difference may be due to differences in the composition of organic carbon molecules produced by each species that can support different groups of bacteria.

The identity of bacteria co-existing with both diatoms belonged to genera that are often recognized as generally microalgal, and specifically diatom, associated. The most dominant bacterial group with all seven diatom strains reported here was the Roseobacter-clade (Rhodobacteraceae). Members of the Rhodobacteraceae have been shown to associate with diatoms in culture and in the field (Grossart et al., 2005; Guannel et al., 2011; Sison-Mangus et al., 2014; Baker and Kemp, 2014; Amin et al., 2015). In addition, they have also been shown to interact in a variety of ways with phytoplankton. For example, *Sulfitobacter* sp. SA11 has been shown to enhance the growth of the diatom *P. multiseries* by converting diatom-derived tryptophan to the hormone IAA. SA11 also provides ammonia to the diatom in exchange for organosulfur compounds such as taurine and dimethylsulfoniopropionate (DMSP). These interactions appear to increase photosynthesis and carbon fixation by the diatom, which may benefit the bacterium (Amin et al., 2015). *Ruegeria pomeroyi* DSS-3 alleviates vitamin B_12_ limitation of the diatom *T. pseudonana* in exchange for organosulfur compounds such as 2,3-dihydroxypropane-1-sulfonate and *N*-acetyltaurine (Durham et al., 2015). The interaction between both species appears to involve a range of genes involved in response to external stimuli, lipid and chitin biosynthesis in the diatom (Durham et al., 2017). *Phaeobacter inhibens* attaches to the coccolithophore *Emiliania huxleyi* and initially promotes its growth by converting algal-derived tryptophan to IAA (Segev et al., 2016). However, *P. inhibens* proceeds to kill *E. huxleyi* during later phases of growth by producing the algicidal molecules roseobacticides (Seyedsayamdost et al., 2011). In contrast, *Roseobacter* sp. DG874 significantly inhibits the growth of the dinoflagellate *Gymnodinium catenatum* potentially due to the bacterial ability to lyse algal cells (Bolch et al., 2017).

Analysis of the 21 Rhodobacteraceae OTUs found with both diatoms shows that only five were common to both diatoms (**Figure 4A**). Most OTUs exhibited variation across strains and time of culturing in the laboratory (**Figure 4B**), despite being highly conserved at the genus level. Such variations at the OTU level likely reflects different metabolic potential for these bacteria and it is not clear how these variations are reflected in interactions with the diatom. It is noteworthy that cocultures of A3 with two *Alteromonas* spp. that have near identical 16S rDNA gene (AGSF14, AGSF23) have different effects on the growth rate of A3 (**Table 2** and **Figure 7**). Interestingly, two OTUs were found consistently with all seven strains and across time. Of these, Rhodobacteraceae_otu A showed strong nucleotide identity to *Shimia* and *Nautella* species, including four strains that we isolated from *A. glacialis*. These findings suggest that our *Shimia* and *Nautella* isolates may represent a good model to examine diatom–bacteria interactions given that they are closely related to an OTU that was found consistently with two diatom species across seven strains. Interestingly, two *S. marina* strains (AGSF11, AGSF28) showed no effect on the growth rate of A3 while a *Nautella* strain (AGSF5) enhanced the growth rate of A3 relative to axenic control (**Table 2** and **Figure 7**). Given that these cocultures were conducted in optimal media under no nutrient limitations it is likely that these bacteria interact with the diatom under special conditions (i.e., nutrient limitation or environmental disturbance).

*A. glacialis* and *N. longissima* isolates also harbored a significant number of reads that belonged to *Neptuniibacter* (**Figure 3**), a genus of γ-proteobacteria that has been shown to be a major player in response to phytoplankton-derived organic matter during blooms (Beier et al., 2014). *Neptuniibacter* reads have also been detected in cultures of toxigenic diatoms (Guannel et al., 2011) and close relatives have been shown to significantly contribute to vitamin B_12_ production in the Southern Ocean sea ice edge where diatom communities dominate (Bertrand et al., 2015). Members of the same family have also been shown to closely associate with blooms of the haptophyte *Phaeocystis antarctica* where they appear to also contribute to vitamin biosynthesis (Delmont et al., 2015).

Other notable OTUs found in both diatoms were unclassified Flavobacteriaceae (**Figure 3**), which contain several members that have been shown to be algicidal to diatoms (Paul and Pohnert, 2011; van Tol et al., 2017). All cultures at 20 days contained one single OTU that belonged to *Mesorhizobium*, which persisted over time in *A. glacialis* but disappeared after 20 days in *N. longissima* (**Figure 3**). *Mesorhizobium* are nitrogen-fixing rhizobial symbionts that provide ammonia to legumes in exchange for organic carbon. Interactions between both taxa involve the production of IAA by the bacterium to facilitate engulfment of bacterial colonies by the plant root hairs (Laranjo et al., 2014). Two members of *Mesorhizobium* have been previously recovered from single-cell diatom isolates from station ALOHA (Baker and Kemp, 2014), suggesting they may be an overlooked components of diatom consortia. It remains to be seen whether these bacteria also fix nitrogen in seawater and whether their interactions with diatoms resemble interactions with plants in soil environments. It is noteworthy that in our sequencing efforts, we attempted to amplify 16S rDNA of archaea; however, we were not able to detect any genuine archaeal sequences suggesting no archaea associate with either diatoms.

*A. glacialis* is a blooming, cosmopolitan diatom that has been isolated from the coastal regions of every continent, including tropical, subtropical and temperate zones (Körner, 1970; Rao, 1969; Choudhury and Panigrahy, 1989). Several studies highlight its dominance in surf zones, where *A. glacialis* is believed to be a major producer of dissolved organic carbon (Lewin and Norris, 1970; Karentz and Smayda, 1984; Du Preez et al., 1989). Several studies also report that bacteria exhibit chemotaxis toward *A. glacialis* exudates (Riquelme and Ishida, 1988) and that some *A. glacialis* strains cannot be grown without specific bacteria (Riquelm et al., 1988). Together, these observations suggest that *A. glacialis* is an important phytoplankton species that interacts with bacteria and likely plays an important role in carbon cycling. Therefore, we focused further efforts on examining whether *A. glacialis* growth is controlled by bacteria.

The varied response of *A. glacialis* A3 to different bacterial isolates highlights the complexity of algal–bacterial interactions. We were only able to cultivate bacteria that belonged to the *Alteromonadaceae* and *Rhodobacteraceae* families (*Alteromonas*, *Shimia*, and *Nautella*) (**Figure 5**). Most *Alteromonas* isolates had no significant effect on A3 with the exception of *A. macleodii* strain AGSF14. Variable responses between diatoms and bacteria across strains has been documented before (Amin et al., 2015), though the mechanisms of why these variations occur are not understood. *Nautella* sp. AGSF5 also enhanced the growth rate of A3 (**Figure 7G**). Bacteria belonging to *Nautella* have been shown to confer resistance to the diatom *Chaetoceros tenuissimus* against viral infection, suggesting they may have a role in algal health and growth (Kimura and Tomaru, 2014).

The consistent growth response of axenic A3 to several bacteria that were previously isolated from other pennate diatoms suggests that the mechanisms of interactions between these bacteria and their original diatom hosts are similar to their interactions with A3. These observations may suggest that diatoms and bacteria interact using common sets of genes and molecules that can be complemented with different hosts or bacteria. For example, *Sulfitobacter* sp. SA11 enhances the growth rate of *P. multiseries* by 19–35% by producing the hormone IAA (Amin et al., 2015). In cocultures reported here, *Sulfitobacter* sp. SA11 enhances the growth rate of A3 by 22% relative to axenic control (**Figure 8A** and **Table 1**), in line with experiments with *P. multiseries*. This observation also suggests that other growth enhancing bacteria, e.g., *Ruegeria* sp. AGSA97 (**Figure 8B**) may use the same mechanism to enhance diatom growth. Likewise, *C. atlanticus* SA60 inhibits the growth of several diatoms including its original host, *P. multiseries*, by ≥ 53%, *P. fraudulenta* by 34%, *T. oceanica* by 15% and *T. pseudonana* by 33% (van Tol et al., 2017). In cocultures reported here, *C. atlanticus* SA60 inhibits the growth rate of A3 by 14% (**Figure 8C** and **Table 1**). The mechanisms of inhibition in A3 may be similar to those reported previously.

Our results suggest that diatoms maintain a stable microbiome even under laboratory culturing conditions and that members of their microbiomes are consistent across different diatom isolates from the same species. These findings strengthen approaches that aim to isolate bacteria from old algal cultures and study their interactions with their algal host. One important caveat to our approach is that the diatom microbiomes were characterized 20 days after isolation. During this time, there may have been changes to the microbiome relative to what was isolated from seawater. Further work is needed to close this gap in knowledge. Since algal-associated bacteria can enhance or inhibit algal growth, these interactions may play an important role in carbon and nutrient cycling. Further work is needed to examine the bacterial diversity across a wide range of phytoplankton species and elucidate their importance to biogeochemistry.

## Author Contributions

GB, MO, and SA designed the experiments. GB, MO, JF, CF, and JK carried out the experiments. GB isolated and identified the diatoms and along with MO characterized their microbial communities. GB, JF, and CF isolated and identified bacteria and conducted coculture experiments. JK conducted the SEM analysis. All authors were involved in writing and reviewing the manuscript.

## Conflict of Interest Statement

The authors declare that the research was conducted in the absence of any commercial or financial relationships that could be construed as a potential conflict of interest.

## Footnotes

1 https://blast.ncbi.nlm.nih.gov/Blast.cgi

2 http://hannonlab.cshl.edu/fastx_toolkit/index.html
